# The influence of growth hormone on pediatric body composition: A systematic review

**DOI:** 10.3389/fendo.2023.1093691

**Published:** 2023-02-09

**Authors:** Alessandro Ferruzzi, Massimiliano Vrech, Angelo Pietrobelli, Paolo Cavarzere, Nicoletta Zerman, Alessandra Guzzo, Carl-Erik Flodmark, Giorgio Piacentini, Franco Antoniazzi

**Affiliations:** ^1^ Department of Surgical Science, Dentistry, Gynecology and Pediatrics, Pediatric Unit, Verona University Medical School, Verona, Italy; ^2^ Pennington Biomedical Research Center, Louisiana State University (LSU) System, Baton Rouge, LA, United States; ^3^ Department of Surgical Science, Dentistry, Gynecology and Pediatrics, University of Verona, Verona, Italy; ^4^ Section of Clinical Biochemistry, Department of Neurosciences, Biomedicine and Movement Sciences, University of Verona, Verona, Italy; ^5^ Department of Clinical Sciences in Malmö, Lunds University, Lund, Sweden

**Keywords:** growth hormone, body composition, growth hormone deficiency, fat mass, lean body mass

## Abstract

**Background:**

Growth hormone (GH) affects metabolism and regulates growth in childhood. The most prominent feature of GH deficiency (GHD) in children is diminished height velocity that eventually leads to short stature. In adult-onset GHD, lean body mass (LBM) is reduced, and visceral fat mass (FM) increased. Beneficial effects of GH treatment on body composition in adults with GHD, including an increase in muscle mass and a decrease in FM, are well established. Relatively few studies have investigated the effects of GH treatment on the body composition of pediatric patients with idiopathic or hypothalamic-pituitary disease-associated GH deficiency. This systematic review aimed to summarize available evidence relating to the effects of GH treatment on body composition in children with GHD.

**Methods:**

The PubMed, Science Direct, Cochrane Trials, and Embase databases, were searched with keywords including “GH”, “body composition”, “children”, and “growth hormone” for English-language articles, published between January 1999 and March 2021. Two reviewers independently evaluated the search results and identified studies for inclusion based on the following criteria: participants had a confirmed diagnosis of GHD (as defined in each study); participants were pediatric patients who were receiving GH or had stopped GH treatment, regardless of whether they were pre- or post-pubertal; the intervention was recombinant human GH (rhGH; somatropin); and outcomes included changes in body composition during or after stopping GH therapy. Data extracted from each study included study quality, study sample characteristics, study interventions, and body composition. Data on fat-free mass and LBM were combined into a single category of LBM.

**Results:**

Sixteen studies reporting changes in body composition (i.e., FM and LBM) associated with GH treatment in children with GHD were identified and included in the review. Collectively, these studies demonstrated that FM decreased, and LBM increased in response to GH replacement therapy.

**Conclusion:**

Despite study limitations (i.e., potential effects of diet and physical activity were not considered), we concluded that a periodic body composition assessment is required to ensure that a satisfactory body composition is achieved during GH replacement therapy in children with GHD.

## 1 Introduction

Growth hormone (GH) and its effector insulin-like growth factor 1 (IGF-1) are needed to regulate growth in childhood and body composition (1–3). Approved indications for GH replacement include short stature associated with Turner, Noonan and Prader-Willi syndromes (PWS), small size for gestational age, renal failure, idiopathic short stature, short stature homeobox-containing gene (SHOX) deficiency, and GH deficiency (GHD) (1).

The most prominent feature of GHD in children is diminished height velocity, but problems associated with GHD extend beyond decreased linear growth in children (4, 5). For example, individuals who develop GHD in adulthood experience a decrease in lean body mass (LBM), and an increase in visceral fat mass (FM) (6). Patients with childhood-onset GHD that persists into adulthood have more severe clinical manifestations than patients with adult-onset GHD, including lower LBM and higher FM (7).

GH activity shows a regulation of body composition starting in the tissue progenitor cells. Before puberty GH promotes skeletal and LBM growth and differentiation, while later in life GH is involved in the regulation of body composition (8). The effects of GH treatment on body composition in adults with GHD has been explored extensively in the literature, and potential benefits have been reported in terms of an increase in LBM and a decrease in FM (9). The effects of GH on body composition have also been investigated in specific pediatric conditions, such as PWS (10). Relatively few studies have investigated this effect on pediatric patients with GH deficiency that is either idiopathic or a result of hypothalamic-pituitary disease.

In 1973, Collipp and colleagues published the first study investigating body composition changes in pediatric subjects receiving GH (11), but more recently it has begun to be explored more extensively. The techniques used to assess body composition have changed over the years, evolving from the measurement of triceps skinfold thickness (ST) in the first studies to dual energy X-ray absorptiometry (DXA), currently the most reliable and widely used method of determining body composition (12, 13), facilitating the assessment of both FM and LBM (14).

This systematic review summarizes the available evidence relating to the effects of GH treatment on body composition in children with a confirmed diagnosis of GHD treated with GH. Given that both GHD and high FM increase the risk of metabolic disturbances such as dyslipidemia and insulin resistance (8, 15), we also considered the effects of GH treatment on plasma lipid and insulin levels when these were reported in studies included in the review.

## 2 Methods

The methodology of this systematic review is consistent with the Preferred Reporting Items for Systematic Reviews and Meta-Analyses (PRISMA) statement (16).

### 2.1 Study selection and data extraction processes

A comprehensive search of all English-language articles analyzing the effects of GH treatment on the body composition of pediatric patients with a confirmed diagnosis of GHD was conducted. We searched several electronic databases (PubMed, Science Direct, Cochrane Trials, Embase) with the keywords “GH”, “body composition”, “children”, “pediatrics”, “growth hormone”, “growth hormone deficiency”, “growth hormone treatment”, “growth hormone supplementation”, “body composition”, “fat mass”, and “lean body mass” to identify relevant studies published between 1 January 1999 and 31 March 2021. The bibliographies of related articles were also searched to identify any additional published references relevant for inclusion in the review.

Search results were exported into the reference manager software “Rayyan QCRI”. Two reviewers (AF and MV) working independently and blindly considered the potential eligibility of each of the titles and abstracts identified after executing the search strategy. They then evaluated the full-text versions of all potentially eligible studies and extracted data from the references. Disagreements were resolved by a third reviewer (AP).

Data extracted from each study were: 1) study quality (e.g., study design, outcomes, and statistical analyses); 2) study sample characteristics (e.g., age, sex, and puberty status); 3) study interventions (e.g., dosage and length of GH therapy, retesting for GHD); and 4) data on body composition (e.g., LBM or fat-free mass, or FM). Because the terms LBM and fat-free mass are typically used interchangeably in scientific literature, we combined data on fat-free mass and LBM into the single category of LBM.

### 2.2 Study eligibility criteria

Studies were included when they fulfilled the following criteria:

• Participants had a confirmed diagnosis of GHD (as defined in each study);
• Participants were pediatric patients who were receiving GH or had stopped GH treatment, regardless of whether they were pre- or post-pubertal;
• Intervention was recombinant human GH (rhGH; somatropin);
• Outcomes included changes in body composition during or after stopping GH therapy.

## 3 Results

**Figure 1:**
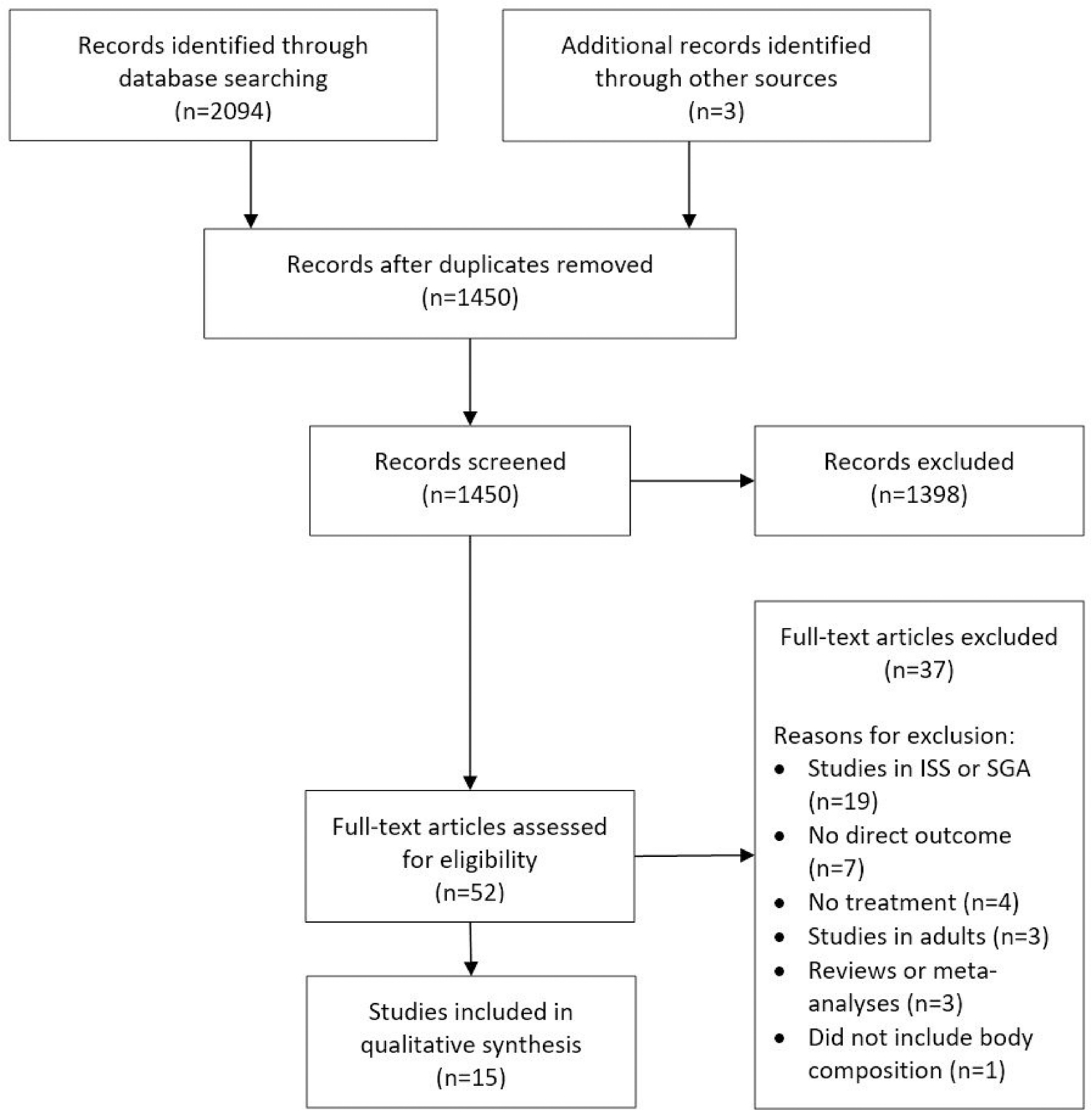
PRISMA flow diagram. This figure is an adaptation from “Preferred Reporting Items for Systematic Reviews and Meta-Analyses: The PRISMA Statement.” by Moher D, Liberati A, Tetzlaff J, Altman DG, The PRISMA Group (2009), and is used under a CC BY-NC-SA 4.0 license. ISS, idiopathic short stature; SGA, small for gestational age.

We identified a total of 2,094 records by searching electronic databases (**Figure 1**). After duplicates were removed, a total of 1,450 records were considered for inclusion. After the first screening, based on title and abstract, 52 full-text articles were selected for assessment of eligibility. After this second screening process, a total of 15 articles were considered eligible for this review (17–31).

Included studies enrolled over 500 pre- or post-pubertal pediatric patients with GHD (**Tables 1** and **2**).

**Table 1:** Studies that evaluated body composition at baseline and during rhGH treatment in children with GHD.

Study	Number of patients with GHD	Mean age (pre/post-pubertal)	Follow up point during treatment (years)	Body composition assessed by	Conclusion
R. Kuromaru (25)	49	10.6 (pre)	3	BIA	↓ FM ↑ LBM
H. Matsuoka (26)	10	11.5 (pre)	1	DXA	↓ FM
T. Sas (29)	22	3–11 (pre)	6	ST	↓ FM
J. N. Roemmich (28)	6	11.4 (pre)	1	DXA	↓ FM (–4.7%) ↑ LBM
I. M. van der Sluis (31)	59	8.3 (pre/post)	6	DXA	↓ FM (after 1 year)/= FM ↑ LBM
P. V. Carroll (18)	12	NA (post)	1	DXA	= FM (–1.4%) ↑ LBM (+1.7 kg)
W. Högler (22)	20	9.4 (pre)	2	DXA	↓ FM (–6%) ↑ LBM (+7.1 kg)
N. Mauras (27)	11	NA (pre)	2 months	DXA	↓ FM (–3.7%) ↑ LBM (+ 2.3 kg)
H. Gleeson (21)	9	NA (pre)	2	II	= FM = LBM
R. Decker (20)	39	7.1 (pre)	2	DXA	↓ FM ↑ LBM
V. Khadilkar (24)	49	9.3 (pre)	1	DXA	↓ FM (–3.3%) ↑ LBM (+3.2 kg)

BIA, bioelectrical impedance analysis; DXA, dual energy X-ray absorptiometry; FM, fat mass (mean difference in % of body weight, where applicable); GHD, growth hormone deficiency; II, infrared interactance; LBM, lean body mass (mean difference in kg, where applicable); NA, not available; rhGH, recombinant human growth hormone; ST, skinfold thickness.

↓, endpoint decreased; ↑, endpoint increased; =, no change shown in endpoint.

**Table 2:** Studies that evaluated body composition after rhGH treatment suspension in children with GHD.

**Study**	**Number of patients with GHD**	**Mean age (pre/post-pubertal)**	**Follow up post-treatment (years)**	**Body composition assessed by**	**Conclusion**
F. J. Cowan (19)	11	17 (post)	1	DXA	↑ FM (+7.8%) = LBM (–1 kg)
G. Johannsson (23)	21	19 (post)	2	DXA	↑ FM (+6.7%) ↓ LBM
M. Tauber (30)	91	(post)	1	DXA	↑ FM ↓ LBM
G. Binder (17)	8	16.7 (post)	0.5	DXA	↑ FM (only in persistent GHD)/= FM ↓ LBM (only in persistent GHD)/= LBM (–0.5 kg)

DXA, dual energy X-ray absorptiometry; FM, fat mass (mean difference in % of body weight, where applicable); GHD, growth hormone deficiency; LBM, lean body mass (mean difference in kg, where applicable); rhGH, recombinant human growth hormone.

↓, endpoint decreased; ↑, endpoint increased; =, no change shown in endpoint.

### 3.1 Baseline endocrine evaluation

The standard insulin tolerance test (ITT) or arginine-ITT was used for the evaluation of GH status and diagnosis of GHD in six studies (18, 20, 23, 25, 26, 30). Additional tests included the clonidine-betaxolol test (30), and the glucagon stimulation test (18). Six studies did not specify which stimulation tests were used (19, 21, 22, 27–29). The diagnosis of GHD was based on a peak GH value of <10 ng/mL in eight studies (17, 20, 22, 24–27, 29), <5 ng/mL in one study (31), and <3 ng/mL in another (18). The maximum GH concentration limit required for a diagnosis of GHD was not specified in five studies (19, 21, 23, 28, 30).

In all but three of the studies (26, 27, 29), information on additional pituitary hormone deficiencies was given. Most patients had idiopathic GHD, although multiple pituitary hormone deficiencies and other diseases were also encountered.

### 3.2 Treatment with rhGH

The initial rhGH dose ranged from 0.02 to 0.042 mg/kg per day. In only one study was the dose personalized from the start (20), ranging between 0.017 and 0.1 mg/kg per day.

### 3.3 Body composition

In eleven studies, body composition was assessed before and during rhGH treatment at different intervals ranging from 2 months to 6 years (**Table 1**) (18, 20–22, 24–29, 31). Six of these studies had a follow-up period greater than 1 year. In four studies, body composition was assessed at the end of therapy and reassessed 0.5–2 years after treatment had stopped (**Table 2**) (17, 19, 23, 30).

Body composition was evaluated using total body DXA in 12 studies (17–20, 22–24, 26–28, 30, 31). The remaining studies used near-infrared interactance (II), bioelectrical impedance analysis (BIA), or ST (21, 25, 29).

DXA is reported to be the best currently available technique for measuring body composition (13, 14); however, LBM data may not be accurate because DXA cannot distinguish between body cell mass and water (32–34).

Most studies concluded that rhGH therapy has beneficial effects on body composition by reducing FM and increasing LBM. There was only one study in which no significant changes in body composition were observed during treatment (**Table 1**) (21). Four studies that analyzed body composition trends after the end of treatment revealed decreases in LBM and increases in FM (**Table 2**), particularly in patients with persistent GHD (17, 19, 23, 30).

### 3.4 Fat mass

**Figure 2:**
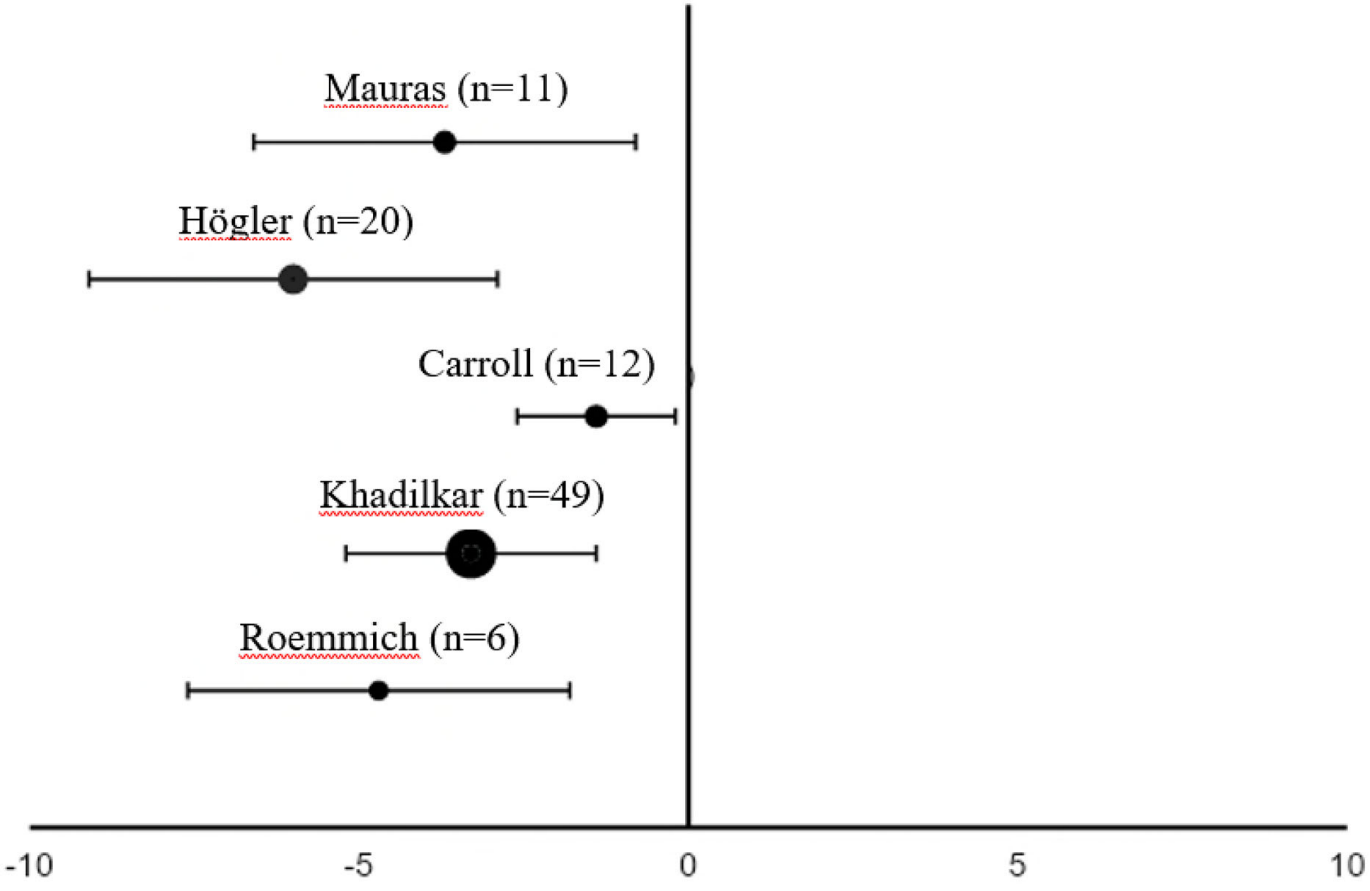
Differences in fat mass (mean % of body weight with 95% confidence interval) from the start to 1 year^a^ of rhGH replacement in pre-pubertal patients^b^ with GHD (18, 22, 24, 27, 28). ^a^Two months of replacement in Mauras et al. ^b^Carroll et al. enrolled a post-pubertal population. GHD, growth hormone deficiency; rhGH, recombinant human growth hormone.

**Figure 3:**
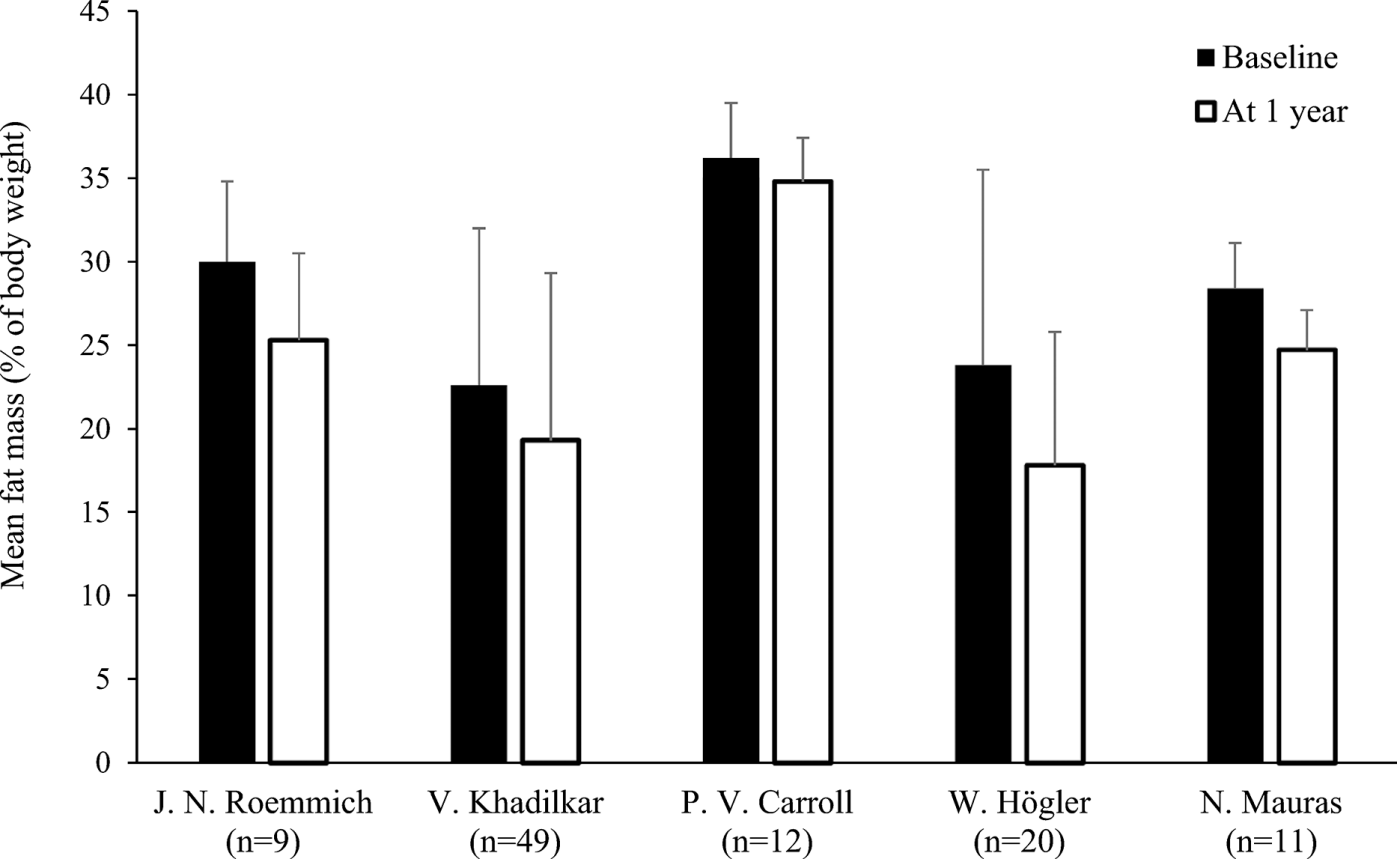
Fat mass (mean % of body weight with standard deviation) at baseline and after 1 year^a^ of rhGH replacement in pre-pubertal patients^b^ with GHD (18, 22, 24, 27, 28). ^a^Two months of replacement in Mauras et al. ^b^Carroll et al. enrolled a post-pubertal population. GHD, growth hormone deficiency; rhGH, recombinant human growth hormone.

**Figure 4:**
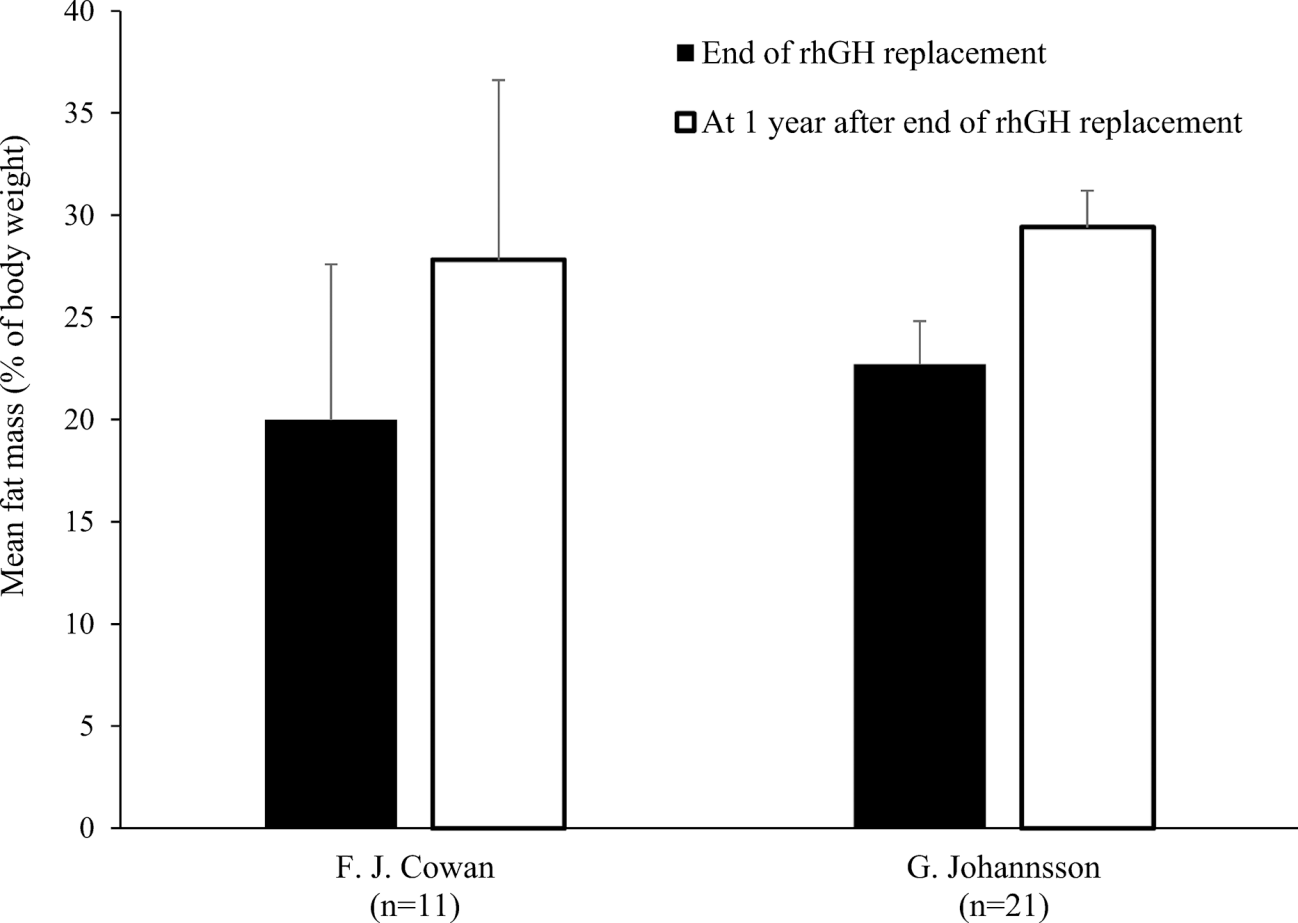
Fat mass (mean % of body weight with standard deviation) at the end of rhGH replacement and 1 year later in pre-pubertal patients with GHD (19, 23). GHD, growth hormone deficiency; rhGH, recombinant human growth hormone.

Only two studies out of eleven found no significant decrease in FM during rhGH replacement therapy (**Table 1**) (18, 21). van der Sluis and colleagues specified that the decrease was significant after 1 year of treatment but not during later years (31). Three of the four studies that evaluated changes in body composition after cessation of rhGH therapy reported increases in FM in the overall study populations (19, 23, 30), while significant increases in FM in the remaining study only occurred in the subgroups with persistent GHD (**Table 2**) (17). Results from studies reporting changes in FM as a percentage of body weight during rhGH therapy and after treatment had stopped are depicted in **Figures 2**–**4**. Overall, there was strong evidence of a decrease in FM during chronic rhGH treatment, with cessation of treatment almost invariably resulting in an increase in FM, particularly in patients with persistent GHD.

### 3.5 Lean body mass

**Figure 5:**
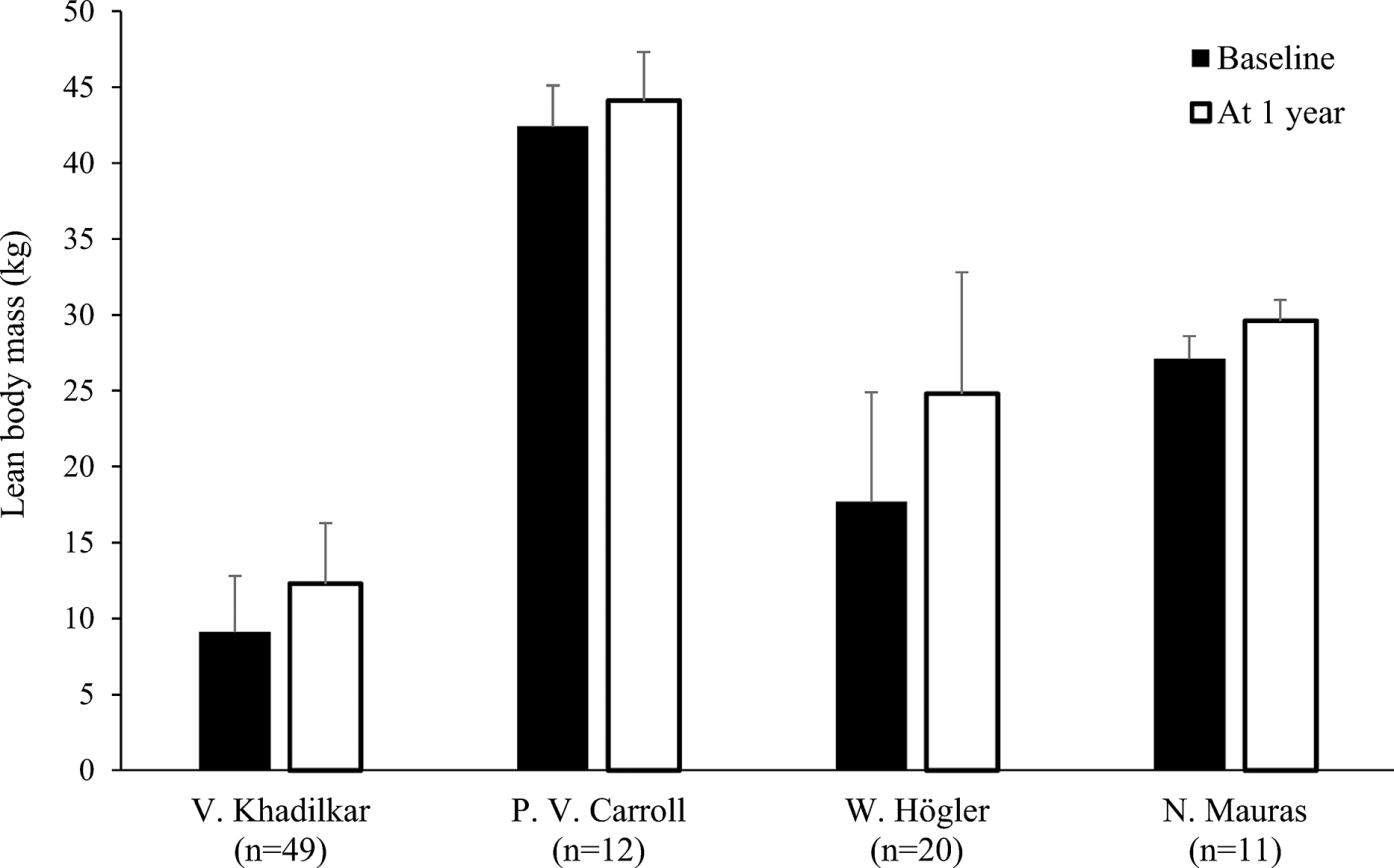
Lean body mass (mean kg with standard deviation) at baseline and after 1 year^a^ of rhGH replacement in patients^b^ with GHD (18, 22, 24, 27). ^a^Two months of replacement in Mauras et al. ^b^Carroll et al. enrolled a post-pubertal population. GHD, growth hormone deficiency, rhGH, recombinant human growth hormone.

**Figure 6:**
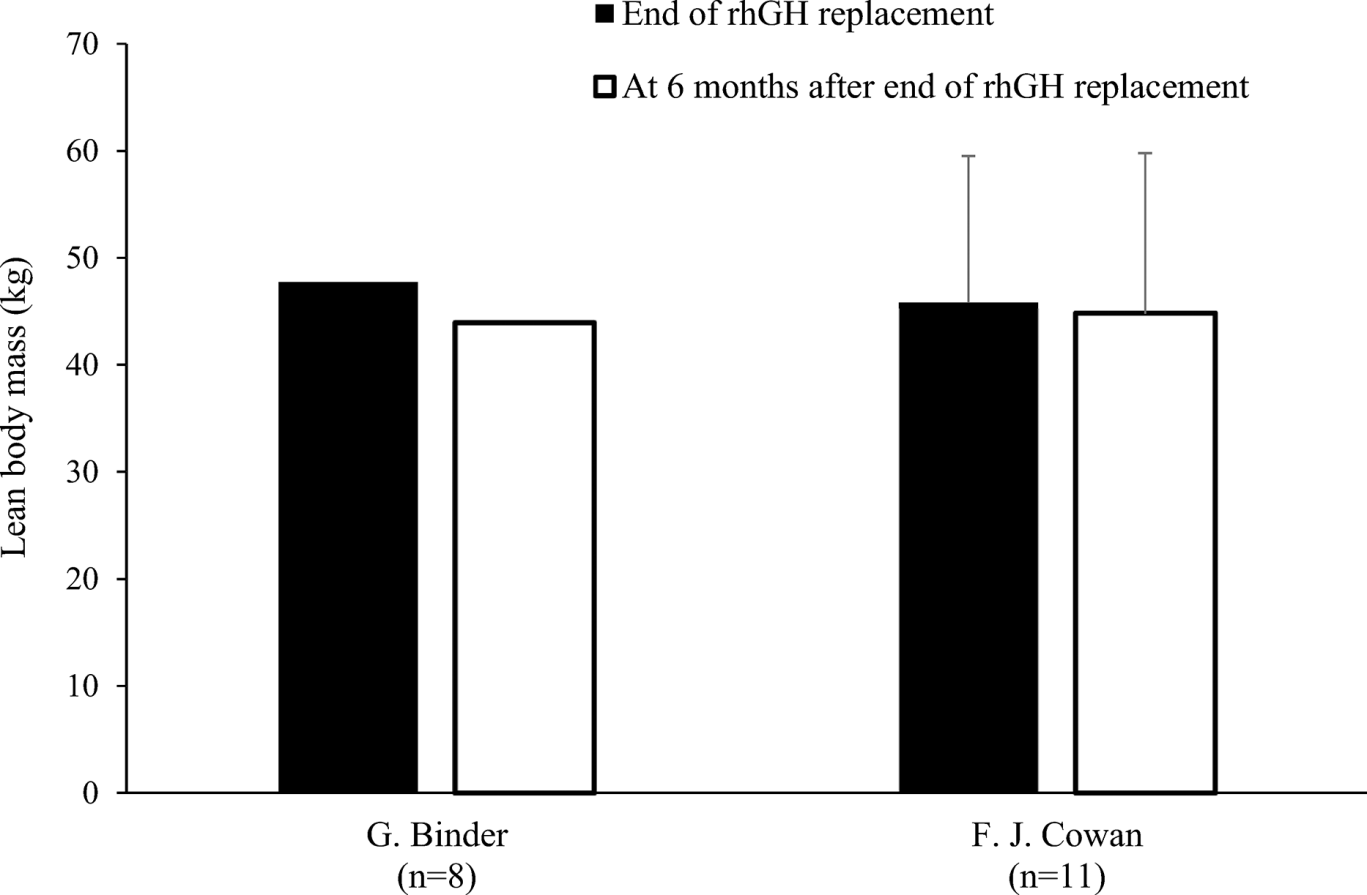
Lean body mass (mean kg with standard deviation [if available]) at the end of rhGH replacement and 6 months later in patients with GHD^a^ (17, 19). ^a^Binder et al. enrolled patients with severe disease (stimulated growth hormone <16 ng/mL and by insulin-like growth factor-1 <–1.90 standard deviation score). GHD, growth hormone deficiency; rhGH, recombinant human growth hormone.

Nine studies determined LBM at baseline and during rhGH treatment (**Table 1**) (18, 20–22, 24, 25, 27, 28, 31). In eight of these studies, there was a statistically significant increase in LBM during rhGH supplementation, as measured with a bioelectrical impedance analyzer (25) or DXA (18, 20, 22, 24, 27, 28, 31). In the remaining study, there was no significant change in LBM, as measured using II (21). Two of the studies that evaluated changes in body composition after cessation of rhGH therapy reported decreases in LBM in the overall study population (23, 30), while significant decreases in LBM in another of these studies only occurred in the subgroups with persistent GHD (**Table 2**), as was also the case for increased FM (17). In the remaining study, there was no significant change in LBM 1 year after cessation of rhGH (19). Results from studies reporting changes in LBM in kg during or after rhGH therapy and after treatment had stopped are depicted in **Figures 5** and **6**. Overall, there was moderate evidence for an increase in LBM during chronic rhGH treatment, as well as a decrease in LBM after discontinuation of rhGH, but this was mostly evident in subjects with persistent GH deficiency.

### 3.6 Plasma lipid and insulin profiles

Nine studies assessed the effects of rhGH replacement on plasma lipid and/or insulin profiles (**Table 3**) (18, 20, 21, 23, 24, 27–29, 31). Five of these studies assessed the effects of rhGH replacement on glucose and insulin levels (18, 20, 23, 27, 30).

**Table 3:** Effects of rhGH therapy on lipid profiles and insulin metabolism in children with GHD (reported differences are intended as statistically significant).

Study	Years of treatment	TC	HDL	LDL	FFA	TG	Insulin	HbA1c	HOMA index
N. Mauras (27)	2 (months)		=	=	=	↑	↑		
T. Sas (29)	4	↓	=	↓					
J. N. Roemmich (28)	1	↓				=	↑		
I. M. van der Sluis (31)	3		↑	↓	↓	=			
H. Gleeson (21)	1	↓	↑	↓		↓		↑	
R. Decker (20)	2						↑		↑
V. Khadilkar (24)	1	=	↑	=		=			
P. V. Carroll (18)	1	=	=	=		=			=
G. Johannsson (23)	2 (after suspension)		↓	=		↓	↓	=	

FFA, free fatty acids; GHD, growth hormone deficiency; HbA1c, serum glycated hemoglobin; HDL, high-density lipoprotein cholesterol; HOMA, homeostasis model assessment; LDL, low-density lipoprotein cholesterol; rhGH, recombinant human growth hormone; TC, total cholesterol; TG, triglycerides.

↓, endpoint decreased; ↑, endpoint increased; =, no change shown in endpoint.

As shown in **Table 3**, three studies demonstrated a decrease in total cholesterol and low-density lipoprotein (LDL) cholesterol during rhGH replacement (21, 29, 31), while four other studies reported no changes in LDL cholesterol (18, 23, 24, 27). Three studies reported significant increases in high-density lipoprotein (HDL) cholesterol (21, 24, 31), while three others reported no significant changes in HDL cholesterol (18, 27, 29). The results regarding triglyceride levels were particularly heterogeneous.

As also shown in **Table 3**, insulin levels increased significantly during rhGH treatment in three studies (20, 27, 28), and decreased significantly after discontinuation of rhGH in one study (23). Glycated hemoglobin (HbA1c) was evaluated in two studies. In one of these, HbA1c increased to the upper end of the normal range during the first 3 months of treatment from a baseline level that was at the lower end of the normal range (21). In the other of these studies, there was no change in HbA1c after cessation of rhGH (23). Further, one study reported no change in homeostasis model assessment (HOMA) during the first year of rhGH treatment (18), while another study reported a strong increase after 2 years of rhGH replacement therapy (20), with a mean value near the cut off associated with metabolic syndrome (35). Overall, there was moderate evidence for a decrease in insulin sensitivity during chronic rhGH treatment. Insulin sensitivity did not appear to be affected in the long term after cessation of rhGH.

## 4 Discussion

Our systematic review demonstrated that GH replacement in pediatric populations with GHD is effective in decreasing FM while increasing their LBM. In particular, we found that there is strong evidence for a decrease in FM during chronic rhGH treatment. Furthermore, in four studies, the cessation of treatment almost invariably resulted in increased FM (17, 19, 23, 30), which was mostly evident in subjects with persistent GHD (17). We also found that there is moderate evidence for an increase in LBM during chronic rhGH treatment. In three studies, discontinuation of rhGH treatment resulted in decreases in LBM, which were mostly evident in subjects with persistent GHD (17, 23, 30). In only one study, in which II was used to assess body composition in nine pre-pubertal children, there was no significant increase in LBM or a decrease in FM (21).

Six studies diagnosed GHD using the standard ITT or arginine stimulation test (18, 20, 23, 25, 26, 30). Additional tests used in these studies included the clonidine-betaxolol test (30), and the glucagon stimulation test (18). The heterogeneity of GH makes it impossible for a single assay to capture all GH moieties that may be present in biological fluids. Indeed, results vary widely based on which GH assay is utilized. GH immunoassays such as single-antibody radioimmunoassays (RIAs), immunoradiometric assays (IRMAs), dual antibody sandwich-type immunoassays, enzyme-linked immunosorbent assays (ELISAs), and immune functional assays (IFAs) (36) detect immunological epitopes on GH molecules and are the most sensitive assays available, due to the high binding affinity of antibodies (36, 37). All immunoassays require a tracer or readout, which may be radioactive, colorimetric, fluorescent, or chemiluminescent. Although the most commonly used immunoassay is the solid-phase, dual-antibody, sandwich-type assay (36), our review analyzed studies that utilized IRMAs.

Historically, clinical guidelines have recommended several different stimulation tests for the diagnosis of GHD, e.g., ITT, arginine stimulation test, clonidine stimulation test, clonidine-betaxolol test, levodopa stimulation test, GH-releasing hormone (GHRH)-arginine stimulation test, and the glucagon stimulation test; all of which carry their respective advantages and limitations (38). GH levels are measured at different times following drug administration, depending on drug type. Additionally, as no single test is 100% effective in eliciting GH release (39), two GH provocation tests are typically conducted. GH levels typically peak 30–90 minutes after insulin administration or onset of arginine infusion, 30–120 minutes after levodopa administration, 60–90 minutes after clonidine administration, and 120–180 minutes after glucagon administration (38). The definition of a ‘normal’ GH response is somewhat arbitrary; generally, a stimulated GH level >10 ng/mL (>10 mcg/L) is sufficient to rule out classic GHD (40, 41).

In the analyzed studies, subjects’ body composition was primarily examined through DXA or BIA. In clinical research, DXA is considered the ‘gold standard’ for assessing FM, fat-free mass (FFM), and bone mineral density (13, 42). However, DXA requires specialized radiology equipment and it is costly, making it almost impossible to use in routine clinical practice. BIA is more commonly used in routine clinical practice (and in research studies); it is a simple, noninvasive, low-cost device that uses the body’s resistance to alternating current to estimate total body water content (TBW) (43, 44). Earlier BIA systems used single-frequency-BIA (SF-BIA) currents and equations that accounted for weight, height, age and the body’s resistance to current flow. With the recent development of multiple frequency-BIA (MF-BIA), researchers and clinical practitioners can now predict intracellular and extracellular water content independently. These systems also calculate a phase angle, a value corresponding to the intracellular:extracellular resistance ratio to cell membrane-specific resistance. As an indicator of cell membrane function, the phase angle is clinically significant, particularly in nutritional and disease risk assessments (45). The phase angle typically decreases with age and height, and increases with greater FFM (46). Notably, several factors may limit the accuracy of BIA in patients with severe obesity. Many of the predictive equations that have been developed in normal-weight subjects with normal body water distribution may not be accurate in severe obesity states; BIA generally underestimates FM in patients with obesity. Comparison of body composition assessment by DXA and BIA according to body mass index (BMI) is poorly documented. In a recent retrospective study, 3,655 measurements of body composition, assessed by DXA and BIA, were compared (47). It was reported that BIA and DXA methods had higher concordance with FM than with FFM values. In this study, the slight bias, particularly in patients with a BMI between 16–18 kg/m^2^, suggested that BIA and DXA methods are interchangeable at a normal population level, but that BIA underestimated FFM and overestimated FM in patients with a BMI ≥18 kg/m^2^.

Two of the analyzed studies assessed body composition using near-infrared interactance (NIR) (21) and measurement of ST with a skinfold caliper (29). ST is difficult to measure with precision and accuracy without rigorous training. Therefore, there is likely to be considerable inter- and intraobserver error in its measurements. ST measurements can mistakenly assume that subcutaneous fat, measured at one or more selected sites, measures total body fat stores. However, subcutaneous fat at one site may not reflect fat stores at another site, and may not be positively correlated with the amount of visceral fat. NIR relies on the fact that different tissue types absorb light at different wavelengths. Percent body fat and lean tissue mass are then obtained by prediction equations based on areas under the curve from spectrophotometric measurements (48). However, NIR cannot differentiate between bone and muscle mass; thus, a lean body mass measurement using NIR reflects both tissue types. Further, NIR is limited in its ability to accurately measure body composition in patients who are extremely obese.

The ‘gold standards’ for quantifying visceral adipose tissue in normal and obese populations are magnetic resonance imaging and computerized tomography. These imaging techniques quantify visceral adipose tissue more precisely than DXA in normal subjects, with coefficients of variation within 1–3% (49–52). This may be explained by DXA’s limited ability to distinguish visceral fat from peripheral fat in the abdominal region. In contrast, DXA measurements are more precise in obese individuals, as altering the reference lines in DXA analysis software can increase or decrease individuals’ fat compartments more slowly than in a lean individual, resulting in less analyst-initiated variation. The precision of DXA visceral adipose tissue assessments should continue to increase as software becomes more accurate at distinguishing visceral fat from peripheral fat. Currently, there are no other validated methods to differentiate between visceral and peripheral fat in this population.

GH treatment seemed to temporarily alter glucose metabolism. In summary, HbA1c, which is known as a specific marker of impaired glucose metabolism (53), was evaluated in two studies, one in which there was an increase in HbA1c during the first 3 months of rhGH treatment to a level at the upper end of the normal range (21), and the other in which there was no change in HbA1c after cessation of GH treatment (30). Moreover, insulin levels increased significantly during rhGH treatment in three studies (20, 27, 28), and decreased significantly after discontinuation of rhGH in two studies (18, 23). In one of these studies, significant increases in insulin levels and HOMA were also reported during rhGH therapy (20). These changes in glucose metabolism could be a consequence of the antagonistic effect of GH therapy with regard to insulin’s action on peripheral tissues, such as the skeletal muscle, liver, and adipose tissue. GH thereby increases glucose production by the skeletal muscle and liver and decreases glucose uptake by adipose tissue (54). Insulin production is increased to compensate for the increased circulating glucose after GH administration (54). We also noted transient changes in lipid metabolism, although results were conflicting (21, 24, 27–29, 31). Indeed, GH has a crucial role in carbohydrate metabolism by acting actively on gluconeogenesis, hyperinsulinemia, and insulin resistance, because it interferes with insulin intracellular signaling, as well as the GH-induced increase of free fatty acids. GH also reduces both abdominal fat and FM in adult subjects with obesity characterized by lower GH secretion, with fewer GH secretory pulses and shorter half-life duration (8).

In general, our findings indicate that GH influences body composition, and therefore periodic changes in FM and LBM should be assessed in children with GHD receiving rhGH therapy to ensure that a satisfactory body composition is maintained over time. Lipid and glucose profiles should also be monitored during treatment.

If during or after GH treatment, no changes are observed in a patient’s body composition, physicians should assess how well the patient complies with the therapy and the appropriateness of their lifestyle/dietary choices; recommendations for additional interventions or lifestyle modifications should be introduced accordingly.

### 4.1 Comparison with previous reviews

Only a few studies have investigated the effect of GH treatment on body composition in pediatric patients with GHD (idiopathic or in hypothalamic-pituitary disease), while the effects on adults have been extensively explored in the literature. In a review of adult data, Berryman and colleagues pointed out that clinical trials with a total number of 982 obese subjects have demonstrated consistent reductions in FM, primarily visceral adipose tissue, without weight-reducing effects, in response to rhGH administration (55). GH secretion, both in the basal condition and after stimulation tests, is blunted in obesity (34). Low levels of GH are associated with altered body composition and cardiovascular risk factors (34).

The first review of the effects of GH therapy on body composition in pediatric populations was published in 1973 by Collipp and colleagues (11). More recently, it has been explored more widely. In 2020, Passone and colleagues reviewed the effects of GH treatment in patients with PWS (10) and found that GH improved stature, body composition (i.e., reduced FM) and BMI, modifying the disease’s natural history.

Our review updates the evidence base relating to the effects of GH therapy on body composition in pediatric patients with GHD. It is becoming increasingly clear that GH therapy, both in pediatric and adult patients, has important implications for body composition, which is more closely related to the patient’s cardiovascular health, and therefore more important, than a simple change in body weight (56). We also suggested timing and reasons for body composition assessments children with GHD (**Table 4**).

**Table 4:** Suggested timing and reasons for body composition assessments children with GHD.

Time - Body Composition Assessment	Outcome
Time 0 - Before GHD Diagnosis	To avoid false positives^a^
Time 1 - After GHD Diagnosis	To define precise dosage related to lean body mass^b^
Time 2 - During GH Treatment	To evaluate GH treatment and body composition changes
Time 3 - End of GH Treatment	To suggest requirement for therapy in adulthood

GH growth hormone; GHD, growth hormone deficiency.

a GH secretion is often blunted in individuals who are overweight or obese. In these individuals, advanced body composition assessments should be used to avoid false positive GHD results.

b Precise assessments of lean body mass help to decide the most effective GH dose and avoid inaccuracies related to using body weight calculations.

Previous studies have demonstrated that GH secretion, at baseline and after a stimulation test, is lower in patients with obesity (as defined by BMI) (57, 58). However, since BMI cannot differentiate between FM- related weight and lean-mass related weight; more specific and precise assessments of body composition should be utilized. These become particularly important in the diagnostic phase for minimizing the likelihood of an improper GHD diagnosis (**Table 4**).

Although BMI and ST provide low cost and rapid body composition assessment, they primarily rely on calculations that do not account for inter-person variation, e.g., lean vs obese individuals, thereby reducing their overall applicability and accuracy. However, DXA, for instance, which is considered more costly, has been shown to determine FM and LBM almost as accurately as more advanced imaging techniques (e.g., magnetic resonance imaging [MRI] and computerized tomography [CT]) (59). Despite DXA’s disadvantages (see Section 3.3), the assessment tool provides rapid and highly accurate results without the high radiation exposure and patient discomfort or accessibility issues associated with MRIs and CTs (59). In the clinical setting, the pay-off for utilizing slightly more expensive yet precise composition monitoring tools is the ability for clinicians to reach a prompter diagnosis/determine treatment effectiveness with increased certainty. Indeed, Binder and colleagues (17) have demonstrated DXA’s ability to act as an essential marker for the relationship between therapy, hormone deficit, and body composition.

### 4.2 Implications for practice and research

Reduction in FM after GH replacement therapy has been described mostly in visceral FM. Recent studies have indicated that the accumulation of visceral fat, rather than subcutaneous fat, is associated with increased cardiometabolic risk (56). This mechanistic difference may be partially explained by differences in the phenotypes of adipose tissue depots (56). Biologically active molecules, such as pro- and anti-inflammatory cytokines and adipokines, are produced at each local fat depot as an independent endocrine organ. However, subcutaneous adipocytes predominantly produce adiponectin, while visceral adipocytes produce leptin, which has been historically associated with type 2 diabetes, hypertension, and other cardiometabolic risk factors.

Future research in this field should include studies with long-term follow-up that enroll homogenous groups of GH-deficient patients with similar causes and onset of disease, and evaluate important outcomes such as quality of life, cardiovascular risk factors, and proportion of visceral fat. It would be important to divide patients by pubertal status, as there are fundamental changes in their metabolism.

### 4.3 Limitations and strengths of our review

This study has several strengths including the comprehensive literature search and review and the appraisal of the risk of bias (**Table 5**).

**Table 5:** Bias assessment.

Study	Selection bias	Detection bias	Blinding of participants and personnel (performance bias)	Selective reporting (reporting bias)	Incomplete outcome data (attrition bias)
R. Kuromaru (25)	✓	✓	X	✓	✓
H. Matsuoka (26)	✓	✓	X	✓	✓
T. Sas (29)	✓	✓	X	✓	✓
J. N. Roemmich (28)	✓	✓	X	✓	✓
I. M. van Der Sluis (31)	✓	✓	X	✓	✓
P. V. Carroll (18)	✓	✓	X	✓	✓
W. Högler (22)	✓	✓	X	✓	✓
N. Mauras (27)	✓	✓	X	✓	✓
H. Gleeson (21)	✓	✓	X	✓	✓
R. Decker (20)	✓	✓	X	✓	✓
V. Khadilkar (24)	✓	✓	X	✓	✓
F. J. Cowan (19)	✓	✓	X	✓	✓
G. Johannsson (23)	✓	✓	X	✓	✓
M. Tauber (30)	✓	✓	X	✓	✓
G. Binder (17)	✓	✓	X	✓	✓

✓, study is subject to bias; X, study is not subject to bias.

However, several limitations exist. We only found articles with small sample sizes that were, in some cases, heterogeneous by age and by pubertal stage. Moreover, in some studies, methods other than DXA were used to measure body composition (BIA, II, and ST), and there was variability across studies in the stimulation tests used to diagnose GHD. Also, although it is clear that diet and physical activity influence and regulate body composition, the majority of the studies considered in this review did not assess the influence of diet and physical activity on body composition during GH therapy.

## 5 Conclusion

Our review clearly shows that rhGH therapy in children with GHD increases LBM and reduces FM. The main implication of this finding is to emphasize the fundamental importance of performing specific and precise periodic assessments of body composition before, during and after treatment. We strongly recommend avoiding the routine measurement of weight or BMI as a substitute for assessing body composition because these parameters may not be reliable indicators of FM or LBM (60). By adopting the suggestions summarized in **Table 4** physicians could reduce errors in the GHD diagnosis pathway and further improve the body of knowledge pertaining to body composition as an indicator of disease status and the most effective timetable for therapy.

## Data availability statement

The original contributions presented in the study are included in the article/supplementary material. Further inquiries can be directed to the corresponding author.

## Author contributions

AF, MV, AP, and FA contributed to the study conception, writing of first draft, and critical revision and editing of subsequent drafts. AF and MV contributed to the data analysis. PC, NZ, AG, C-EF, and GP contributed to critical revision and editing of subsequent drafts. All authors approved the final version for submission and agree to be accountable for the content of the work. All authors contributed to the article and approved the submitted version.
